# Coronary Artery Disease and Atrial Fibrillation in HighBleeding Risk: Indications and Optimal Antithrombotic Therapy After Percutaneous Left Atrial Appendage Occlusion

**DOI:** 10.3390/jcm15187181

**Published:** 2026-09-16

**Authors:** Alessandro Mazzapicchi, Francesco Gallo, Luca Zanarelli, Francesco Filice, Michele Trichilo, Alfonso Ielasi, Maurizio Tespili, Macarena Grassi, Gennaro Carmine Semeraro, Giuseppe De Luca, Francesco Giannini

**Affiliations:** 1 Catheterization Laboratory and Cardiovascular Interventional Unit, Division of Cardiology, Policlinico San Marco, 24040 Osio Sotto, Italy; alessandro.mazzapicchi@grupposandonato.it (A.M.);; 2 Cardiology Unit, “Ospedale dell’Angelo”, AULSS3 Serenissima, 30174 Mestre, Italy; galfra87@gmail.com; 3 Catheterization Laboratory and Cardiovascular Interventional Unit, Division of Cardiology, IRCCS Ospedale Galeazzi Sant’Ambrogio, 20157 Milan, Italy; 4 Division of Cardiology, Azienda Ospedaliera Universitaria Policlinico Gaetano Martino, University of Messina, 98125 Messina, Italy

**Keywords:** left atrial appendage occlusion, coronary artery disease, bleeding risk, antithrombotic therapy, dual antiplatelet therapy

## Abstract

**Background/Objectives:** Atrial fibrillation and coronary artery disease frequently coexist in patients undergoing percutaneous left atrial appendage occlusion (LAAO), creating a complex therapeutic setting in which prevention of device-related thrombosis, cardioembolic events, and coronary ischemic complications must be balanced against a high or prohibitive bleeding risk. The optimal post-procedural antithrombotic regimen remains uncertain, particularly in patients with previous or recent percutaneous coronary intervention or acute coronary syndrome. **Methods:** This narrative review examines the rationale and current evidence supporting antithrombotic therapy after LAAO and integrates these data with contemporary strategies for abbreviated or de-escalated antiplatelet therapy after coronary intervention. **Results:** Device-related thrombosis occurs predominantly within the first 45–90 days after implantation, corresponding to the period of incomplete device endothelialisation, whereas thrombotic risk after coronary stenting or acute coronary syndrome is similarly greatest during the early phase and progressively declines thereafter. This temporal overlap supports an initially protective regimen followed by early treatment simplification whenever appropriate. We propose a pragmatic framework in which treatment intensity is determined by a global bleeding–thrombotic risk profile incorporating bleeding history, anaemia, comorbidities, frailty, coronary presentation, procedural complexity, and follow-up imaging. The approach includes predefined triggers for rapid de-escalation in the presence of relevant bleeding or haemoglobin decline and escalation when device-related thrombosis is detected. **Conclusions:** Current evidence remains heterogeneous and largely observational, the proposed algorithm should be considered a decision-support framework rather than a prescriptive pathway. Prospective randomised studies are needed to define the safest individualised regimen for patients with concomitant LAAO and coronary artery disease.

## 1. Introduction

Atrial fibrillation is associated with a substantial increase in thromboembolic risk, with the left atrial appendage (LAA) being the main site of thrombus formation in non-valvular atrial fibrillation (NVAF). Percutaneous LAA occlusion (LAAO) has therefore emerged as an alternative strategy for preventing stroke in patients who are not suitable candidates for long-term oral anticoagulation (OAC) therapy or who are at high risk of bleeding [1,2].

Randomised clinical trials and subsequent registry data have demonstrated that LAAO provides comparable stroke prevention to anticoagulation, while potentially reducing the long-term risk of bleeding once adjunctive pharmacotherapy is discontinued [3]. However, the optimal post-procedural antithrombotic regimen remains uncertain and is still evolving. The rationale behind antithrombotic therapy following LAAO differs fundamentally from the long-term anticoagulation therapy employed in cases of NVAF. While OAC therapy for AF aims to prevent thrombus formation within the left atrial appendage, pharmacological therapy after an LAAO procedure is primarily intended to prevent device-related thrombosis (DRT) during the period required for endothelization of the device. DRT is a recognised complication of LAAO that has been linked to an increased risk of stroke and systemic embolism.

Many patients undergoing LAAO also present with coronary artery disease (CAD), requiring antiplatelet therapy due to prior percutaneous coronary intervention (PCI) or acute coronary syndrome (ACS). The presence of both conditions creates a complex therapeutic scenario, as clinicians must balance preventing DRT, ischaemic stroke and coronary events with avoiding major bleedings.

The aim of this review is to analyse the clinical evidence for modern antithrombotic therapies after LAAO, particularly in patients with concomitant CAD. The review will provide a step-by-step algorithm for tailoring therapy to the patient’s risk profile.

## 2. Rationale for Antithrombotic Therapy After LAAO

The primary goal of antithrombotic therapy following LAAO is to prevent DRT, which is defined as the formation of a thrombus on the atrial surface of the implanted device. DRT after LAAO occurs in approximately 3–5% of cases and is associated with a 3–4-fold increased risk of ischaemic stroke or systemic embolism. [4]. In pooled data from the PROTECT AF, PREVAIL, and associated registries (*n* = 1739 patients receiving the Watchman Left Atrial Appendage Closure device), DRT was identified in 3.74% of patients [3].

The pathogenesis of DRT is multifactorial, reflecting the interaction between device-related factors, patient characteristics and procedural aspects. Following device implantation, the atrial-facing surface is initially non-endothelialised, creating a thrombogenic interface that is exposed to circulating blood. During this phase, platelets adhere and activate, and fibrin is deposited, promoting thrombus formation. Over time, progressive endothelialisation of the device surface is expected; however, incomplete or delayed healing may prolong the prothrombotic state.

Device design may also influence thrombogenicity. Differences between plug-type and pacifier-type devices can affect flow dynamics and surface exposure, although definitive comparative data remain limited. The Amulet IDE trial and the SWISS-APERO [4,5] trial reported comparable DRT rates between Amulet and Watchman devices, though the SWISS-APERO trial found significantly lower definite/possible DRT with the Amulet device (3.7% vs. 11.8%) when including CT-based possible DRT.

Patient-related factors play a crucial role: advanced age, renal dysfunction, a history of thromboembolism and a high CHA_2_DS_2_-VASc score are all associated with an increased risk of thrombosis. However, bleeding risk often limits the intensity of antithrombotic therapy, creating a challenging therapeutic balance.

Technical aspects are also implied in DRT risk; device implantation depth is an independent risk factor for DRT; deeper device implantation may leave a large uncovered left atrial appendage area which is strongly associated with a higher risk of DRT [6].

Observational data and registries have shown that patients with DRT may be at significantly higher risk of ischaemic stroke and systemic embolism than those without DRT. This association highlights the importance of prevention and early detection strategies. Furthermore, DRT may necessitate an increase in antithrombotic therapy, often reintroducing anticoagulation in patients who were initially deemed unsuitable for it [7,8]. Evidence shows that DRT predominantly occurs in the early post-procedural phase, typically within the first 45 to 90 days after implantation; this corresponds to the period before the device is almost completely endothelised [8].

This observation has direct therapeutic implications. The early phase represents a high-risk window during which a more intensive antithrombotic strategy may be justified to mitigate thrombus formation. Follow-up imaging using transoesophageal echocardiography (TEE) or cardiac computed tomography (CT) 45–90 days after the procedure is recommended to detect DRT and assess device sealing. However, given the non-negligible incidence of late device-related thrombosis, post-LAAO imaging surveillance should reasonably be extended beyond six months. However, late DRT events have also been reported [4], suggesting that thrombotic risk may persist beyond the initial healing phase, particularly in high-risk patients or in the presence of residual anatomical or device-related issues. This highlights the need for individualized long-term strategies rather than a uniform approach.

Overall, DRT is the main pathophysiological link between LAAO and the requirement for post-procedural antithrombotic therapy. Due to the heterogeneity of patient profiles and the lack of a universally optimal regimen, standardising antithrombotic therapy remains challenging. A risk-stratified approach that balances thrombotic and bleeding risks appears to be the most rational strategy in contemporary clinical practice.

## 3. Evidence on Post-LAAO Antithrombotic Regimens

Historically, the initial post-LAAO antithrombotic strategy has been derived from trials such as PROTECT-AF and PREVAIL. In these studies, patients initially received warfarin plus aspirin for 45 days. This was followed by dual antiplatelet therapy (DAPT) for up to six months, after which they transitioned to lifelong single antiplatelet therapy (SAPT) [3,9]. This regimen demonstrated the non-inferiority of LAAO compared to warfarin in terms of stroke prevention, with a significant reduction in haemorrhagic stroke and major bleeding observed during long-term follow-up.

Due to different patient selection criteria, AT (Anti-Thrombotic) therapy after LAAO has undergone a gradual evolution over time. In European countries, where LAAO is usually performed on patients who cannot tolerate oral anticoagulants (OACs), alternative treatment regimens have been studied. Indeed, the available evidence is characterised by remarkable heterogeneity in post-procedural strategies across clinical trials. In the ASAP study, DAPT was administered for six months, followed by aspirin monotherapy, reducing the risk of stroke and major bleeding over mid- and long-term follow-up [10,11]. In contrast, the AMULET-IDE trial permitted greater flexibility, with patients receiving either DAPT or a combination of aspirin and OAC at discharge at the investigator’s discretion [12]. Similarly, the SWISS-APERO trial involved treating patients with either DAPT or OAC for three months, followed by aspirin monotherapy [13]. Finally in the PRAGUE-17 trial, the vast majority of patients (82.3%) were discharged on DAPT, while only a minority (16%) received OAC [14].

In real-world practice, however, most patients are unable to tolerate a DAPT regimen. In the EWOLUTION registry [15], despite the protocol initially including aspirin and warfarin for 45 days followed by DAPT up to 6 months and then lifelong aspirin, 72% of patients were deemed unsuitable for OAC and were therefore switched to (D)APT or no therapy immediately after WATCHMAN device implantation. At 3 to 6 months, 66% of patients were on SAPT or no therapy, increasing to 84% at the end of follow-up. In line with those indications, a simplified AT therapy after LAAO has started to gain ground. In a cohort of 628 patients who received an LAAO for very high-bleeding-risk conditions, 31% of patients were discharged with a light regimen (SAPT or No Therapy) without increasing the rate of ischemic cerebrovascular events or systemic embolism [16]. Moreover, in a multicentre registry of 1135 patients undergoing LAAO, 35% received SAPT or no therapy after the procedure. There were no differences in terms of survival, stroke, transient ischaemic attack (TIA), peripheral embolisms or DRT. However, there was a reduction in bleeding events (HR 0.61, 95% CI: 0.36–0.99; *p* = 0.049) [17]; these observational findings should be interpreted with caution because of the inherent risk of selection bias and confounding by indication. In clinical practice, simplified regimens or complete antithrombotic discontinuation are generally reserved for patients perceived to be at the highest bleeding risk. Therefore, these data primarily support the feasibility of treatment simplification in highly selected patients and cannot establish its comparative safety or efficacy with the same level of confidence as randomized controlled trials.

Ultimately, a different regimen involving apixaban at a dose of 2.5 mg twice daily for three months has been studied in some observational registries and in a small trial. This regimen has been shown to be associated with favourable results and a reduction in any type of bleeding compared with DAPT (4.6% vs. 28.2%) [18,19].

## 4. Time-Dependent Antithrombotic Strategies: A Unifying Framework Between Pci and LAAO

**Figure 1:**
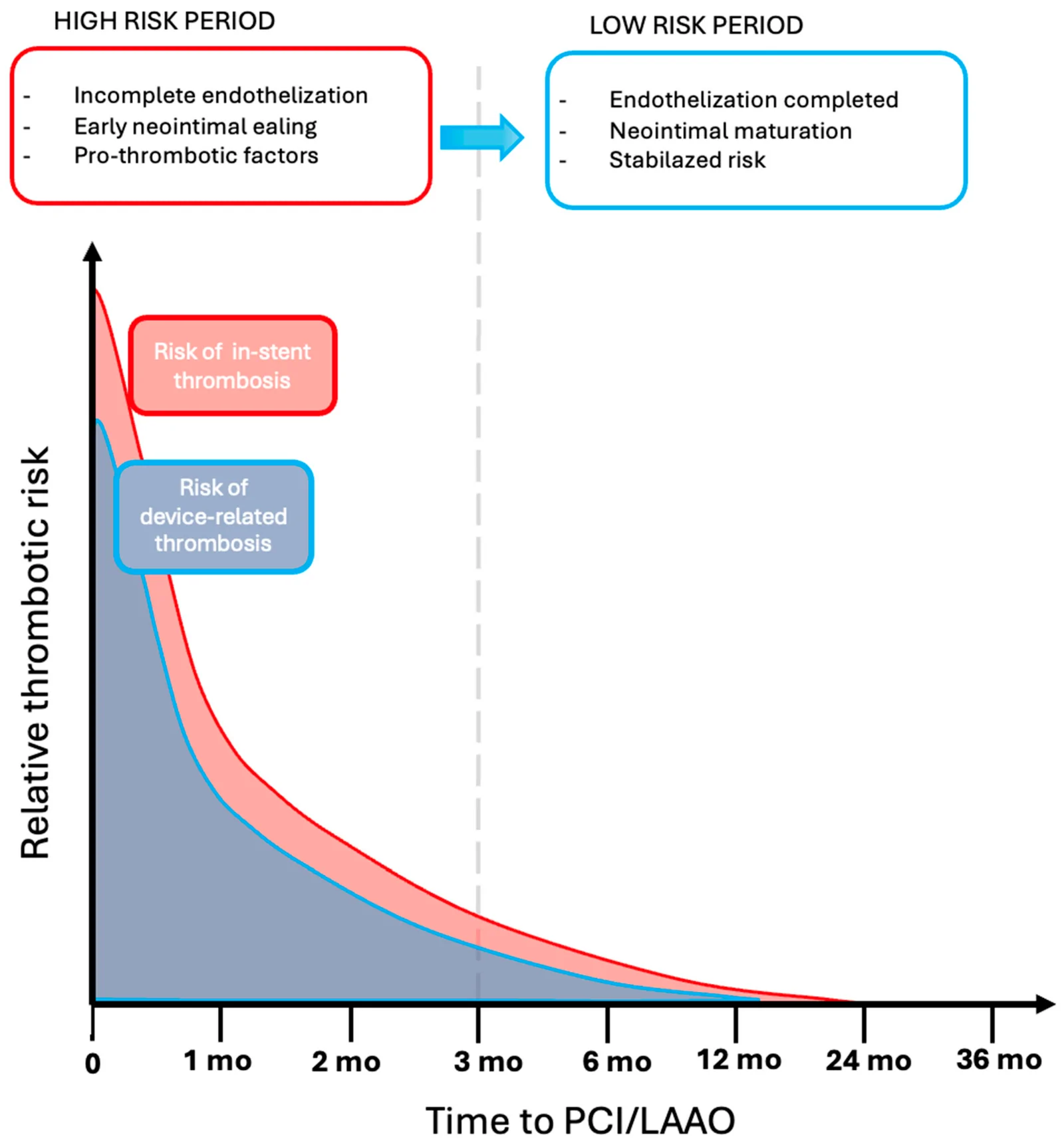
Temporal evolution of thrombotic risk after PCI and LAAO. Schematic representation of the early overlap between stent thrombosis and device-related thrombosis risk, followed by a progressive decline as vascular and device healing occur.

The central concept of this review is that antithrombotic therapy after LAAO should be driven primarily by the temporal evolution of thrombotic risk rather than by fixed treatment durations. This biological framework closely mirrors contemporary PCI practice, where treatment intensity progressively declines as device-related thrombogenicity decreases. The thrombotic risk following cardiovascular procedures is dynamic and time-dependent, rather than static. This principle is well established in the context of coronary disease, where antiplatelet therapy is aimed primarily at preventing stent thrombosis in chronic coronary syndromes (CCS), as well as preventing ischaemic recurrences in acute coronary syndromes (ACS). This involves a front-loaded strategy that targets early thrombotic risk, followed by a gradual reduction in complexity once endothelialisation has been achieved (Figure 1).

### 4.1. LAAO in Chronic Coronary Syndrome Without Revascularization

For patients with chronic coronary syndrome (CCS) who have not undergone a recent PCI, the only relevant thrombotic substrate is the temporary risk of DRT that, as discussed, is time-limited. Real-world data and registries suggest that, once the early post-procedural phase has passed, simplified antithrombotic strategies ranging from SAPT to no therapy in selected cases may be sufficient. This reflects the absence of an ongoing, platelet-driven ischaemic risk in stable coronary disease, particularly in patients selected for LAAO due to a high risk of bleeding. In this context, a logical approach would be to focus on early de-escalation, guided by the understanding that the biological process requiring protection—device endothelialisation—is transient.

### 4.2. LAAO and Pci in Chronic Coronary Syndrome: Managing Overlapping Risks

Although mechanistically different, stent thrombosis and DRT share a remarkably similar biological evolution, with the highest thrombotic vulnerability occurring shortly after device implantation and progressively declining thereafter. This convergence provides a strong rationale for a temporally synchronized antithrombotic strategy.

Evidence from randomized trials has fundamentally reshaped our understanding of DAPT duration after PCI. The traditional paradigm of prolonged DAPT has been progressively challenged by studies demonstrating that shorter regimens are sufficient to protect against early ischemic events while significantly reducing bleeding. Several trials have shown that reducing the duration of DAPT may be a reasonable option, especially in patients with high risk of bleeding. The STOPDAPT-2 trial represents a landmark in this regard, showing that 1-month DAPT followed by clopidogrel monotherapy was not only non-inferior but superior to 12-month DAPT for a composite endpoint of ischemic and bleeding events (HR 0.64), driven primarily by a reduction in bleeding [20]. Similarly, the MASTER DAPT trial extended these findings to high-bleeding-risk populations, demonstrating that discontinuation of DAPT at 1 month resulted in comparable ischemic outcomes but significantly lower bleeding, regardless of procedural complexity [21,22].

A brief consideration should also be given to surgical revascularization. In patients undergoing coronary artery bypass grafting (CABG) in CCS, single antiplatelet therapy is generally sufficient, and the left atrial appendage is often surgically excluded.

Collectively, these data converge on a consistent message: the protective effect of DAPT is concentrated in the early post-PCI phase, whereas prolonged therapy primarily increases bleeding. When translated to the LAAO setting, this evidence supports a unified strategy in which DAPT is maintained during the early 1–3 months, covering both stent thrombosis and DRT. Rapid de-escalation follows, aligning with the completion of endothelialisation processes for both devices. This temporal alignment represents a key conceptual bridge between PCI and LAAO.

### 4.3. LAAO in Acute Coronary Syndrome: Balancing Competing Risks

The challenge becomes even more pronounced in patients presenting with ACS, where ischemic risk is highest, yet bleeding risk is often equally relevant, particularly in those selected for LAAO.

Historically, ACS has been associated with prolonged DAPT regimens. However, contemporary evidence clearly demonstrates that ischemic risk is heavily front-loaded, with the greatest benefit of intensive platelet inhibition occurring within the first 1 to 3 months. Beyond this phase, the relative contribution of bleeding becomes increasingly dominant. The concept of early de-escalation in ACS has further refined this approach.

In the TWILIGHT ACS substudy [23] the benefit of ticagrelor monotherapy, after 3 months of DAPT, was maintained even in patients presenting with NSTE-ACS, reducing clinically meaningful bleeding events, without increasing ischaemic risk as compared with ticagrelor plus aspirin.

Nevertheless, these trials enrolled a mixed population of CCS and ACS patients, with the latter accounting for less than half of the cases. The trials also included a variety of antiplatelet therapies associated with aspirin (i.e., clopidogrel or Ticagrelor) [21,24]. Two trials focusing on the ACS population [25,26] have demonstrated the advantages of an abbreviated DAPT in terms of bleeding. Otherwise, the monotherapy after one or three months was based on ticagrelor, which some patients undergoing LAAO could not tolerate due to a high bleeding risk. Moreover, these studies enrolled a low-risk population resulting in a lower than expected rate of ischemic events [25]; for this reason, the safety of abbreviated DAPT in ACS patients should still be clearly demonstrated. Aspirin withdrawal and clopidogrel monotherapy after one or two months also failed to demonstrate non-inferiority in terms of ischemic events compared with standard DAPT in patients with ACS [27,28].

Notably, most contemporary randomized trials evaluating DAPT strategies have adopted composite endpoints that combine ischemic and bleeding events, often framed as “net clinical benefit”. While this approach improves statistical efficiency, it inherently merges events driven by opposite biological mechanisms and distinct temporal trajectories. As a consequence, such composites may reflect an artificial equilibrium between competing risks rather than a true treatment effect on a unified pathophysiological process.

This limitation becomes particularly relevant when interpreting de-escalation strategies, where reductions in bleeding may outweigh stable or only marginally increased ischemic risk, thereby obscuring the time-dependent rationale that should instead guide antithrombotic therapy.

In the context of LAAO, these considerations acquire even greater relevance. Patients undergoing LAAO are, by definition, those in whom long-term exposure to antithrombotic therapy is problematic due to elevated bleeding risk. Accordingly, the possibility of limiting DAPT duration, implementing early de-escalation, and avoiding prolonged anticoagulation defines a uniquely favourable therapeutic paradigm.

A coherent strategy therefore emerges, particularly in ACS patients undergoing LAAO: an initial phase of intensified antithrombotic therapy, targeting both stent thrombosis and device-related thrombosis, followed by a rapid transition toward simplified regimens aimed at minimizing bleeding exposure.

Importantly, such early treatment simplification should not be interpreted as a strategy supported by robust evidence of equivalent ischemic protection in ACS. Rather, in this highly selected population undergoing LAAO, it represents a clinical compromise driven by the prohibitive bleeding risk associated with prolonged intensive antithrombotic therapy.

These observations reinforce the concept that antithrombotic therapy after both PCI and LAAO should not be interpreted through static endpoints, but rather through a dynamic, biological time-dependent framework that aligns treatment intensity with the evolving biological risk.

Ultimately, having established that thrombotic and bleeding risks evolve dynamically over time, baseline bleeding-risk assessment remains essential to identify patients in whom earlier treatment simplification may be justified.

## 5. A Risk-Stratified Approach: Is HAS-BLED Score Still Valuable?

Although a risk-based approach to antithrombotic therapy after LAAO is conceptually appealing, it is not sufficiently standardised. Of the available tools, the HAS-BLED score remains a pragmatic way of estimating bleeding risk in AF patients treated with OAC. A score of 3 or more indicates a high risk of bleeding and has been shown to confer an approximately three-fold greater risk of major bleeding compared to scores of less than 3. However, it has several limitations: (1) It was developed and validated using warfarin and it is only modestly applicable in the DOAC era. (2) It has only modest discriminative accuracy, similar to other bleeding risk scores. A recent 2025 comparative analysis of 3259 AF patients confirmed that all bleeding risk scores (HAS BLED, ORBIT, DOAC, AF-BLEED) only modestly performed in predicting major bleedings, non major bleeding or intracranial haemorrhage (c-indexes < 0.7), with none demonstrating clear superiority [29].

The LAAO patient population is inherently high-risk, making it challenging to identify individuals who may benefit from a “light” antithrombotic therapy. Otherwise, not all bleeding patients present the same bleeding risk profile; it has been demonstrated that patients undergoing LAAO due to gastrointestinal bleeding are more prone to develop bleeding recurrences, despite antithrombotic therapy downgrading or discontinuation [30]. In the Amplatzer post-market study, which included a total of 1088 patients undergoing LAAO and stratified by CHADS_2_VASC and HAS-BLED, the latter was found to be inaccurate in predicting the risk of fatal bleedings. More patients at lower risk (HAS-BLED < 3) experienced an event (1.3% vs. 0.7%) [31].

Data from large observational cohorts confirm that the incidence of major bleeding increases progressively with higher baseline risk profiles, supporting the biological plausibility of risk stratification [32].

In the field of coronary artery disease, the Academic Research Consortium for High Bleeding Risk (ARC-HBR) has recently defined high bleeding risk as a Bleeding Academic Research Consortium (BARC) 3 or 5 bleeding risk of ≥4% at 1 year. The consortium has proposed 20 consensus criteria for a standardised definition of bleeding risk following PCI. These criteria indicate that an intensive antithrombotic therapy might be poorly tolerated by those patients [33].

A temporal paradigm has been extensively described in the PCI setting, where ischemic risk—particularly stent thrombosis—is highest in the early postoperative period, whereas bleeding risk persists over time.

It is important to note that the biological rationale underpinning these strategies has been clearly elucidated in de-escalation trials. In HOST-REDUCE-POLYTECH-ACS, the benefit of potent antiplatelet therapy was shown to be predominantly confined to the early phase after PCI, whereas continuation of high-intensity therapy during the chronic phase translated mainly into excess bleeding without additional ischemic benefit. This lends further support to the notion of a time-dependent shift in the balance between thrombotic and haemorrhagic risk [34].

When translated to LAAO, the pathophysiological and temporal framework suggests that DRT may follow a time-dependent pattern. Events cluster within the first months after implantation and become progressively less frequent thereafter, reflecting the transient nature of device endothelialisation [4,6]. In this context, a short course of DAPT followed by early simplification is a rational and biologically consistent strategy. It is sufficient to cover the transient phase of the highest DRT risk, while minimising cumulative bleeding exposure in a population that is intrinsically predisposed to haemorrhagic complications.

It is important to note that the relationship between bleeding scores and haemorrhagic risk appears continuous rather than dichotomous. This suggests that bleeding risk should not be interpreted as a binary variable, but rather as a gradient that may guide the intensity and duration of post-procedural antithrombotic therapy.

From a pathophysiological perspective, the fundamental question is whether the transient risk associated with DRT is deemed to exceed the immediate and potentially life-threatening bleeding risk. In such cases, the prevailing approach involves a substantial minimization of antithrombotic exposure, despite the concomitant theoretical increase in thrombotic risk, with the aim of averting catastrophic haemorrhagic events.

## 6. Proposed Algorithm for Post-LAAO Antithrombotic Therapy

In this context, we propose a pragmatic and clinically oriented approach in which bleeding risk becomes the primary determinant of antithrombotic intensity, rather than a secondary modifier.

Routine long-term OAC is not considered the default strategy within the proposed framework, as many patients undergoing LAAO are selected precisely because prolonged anticoagulation is undesirable or contraindicated. However, emerging evidence suggests that short-term low-dose DOAC therapy may represent a reasonable alternative in carefully selected patients without an absolute contraindication to anticoagulation.

In particular, low-dose apixaban has shown encouraging results after LAAO [18,19], although the available evidence remains limited. Accordingly, short-term low-dose DOAC therapy may be considered when the perceived risk of DRT is substantial and bleeding risk remains acceptable, while treatment selection should remain individualized according to the balance between thrombotic and hemorrhagic risk.

Our proposed approach reframes LAAO management as a dynamic process, based on three pillars:

1. Baseline bleeding risk defines initial therapy.
2. Time-dependent thrombotic risk justifies only short-term protection.
3. Continuous monitoring guides real-time adaptation.

The algorithm proposed here does not claim to be definitive, but rather provides a structured method to manage uncertainty in a highly complex population. The proposed algorithm should be interpreted as a pragmatic decision-support framework rather than a prescriptive treatment pathway. Individual clinical judgment remains essential, as patient-specific characteristics, procedural factors, and evolving clinical conditions may justify deviation from the proposed strategy.

**Figure 2:**
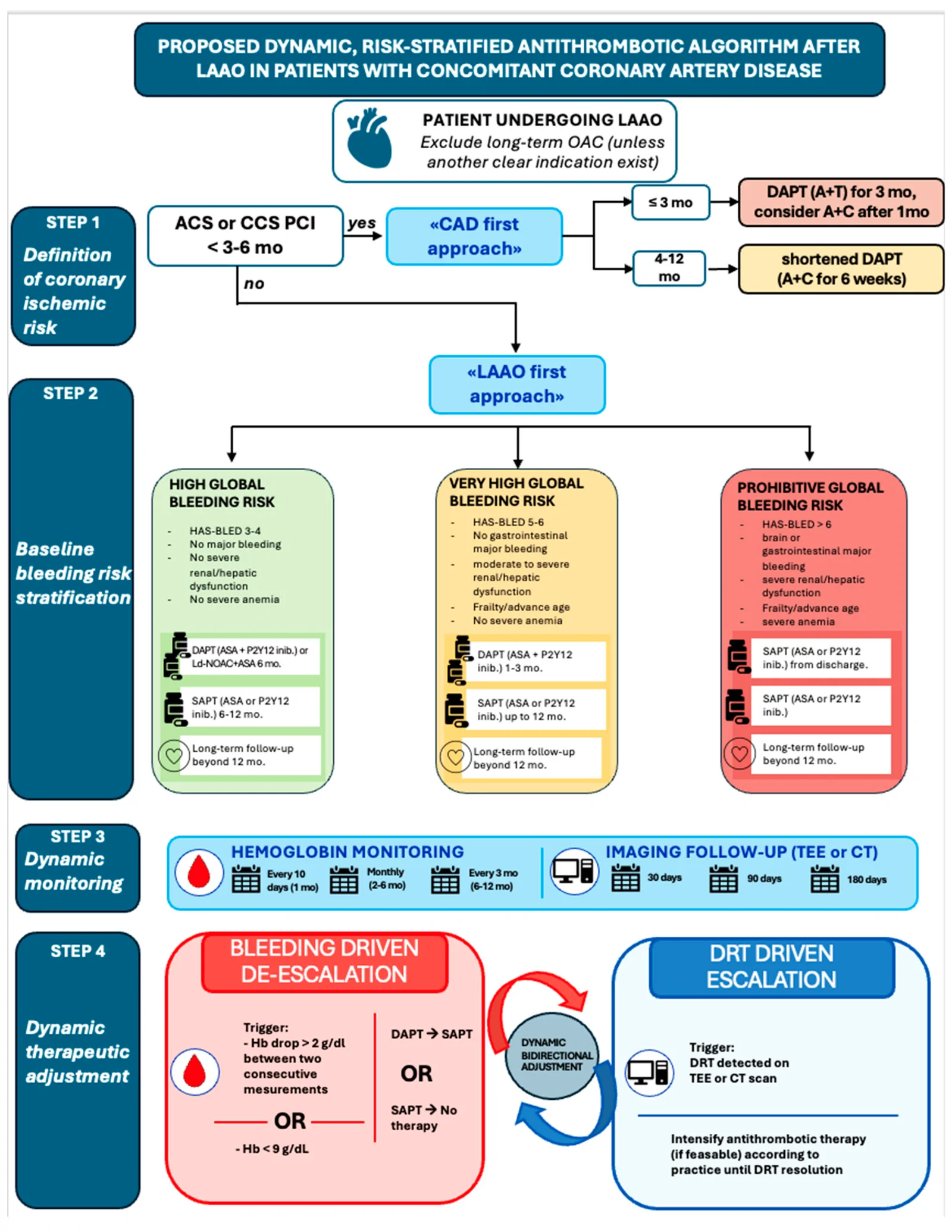
Proposed dynamic, risk-stratified antithrombotic algorithm after LAAO in patients with concomitant coronary artery disease. Antithrombotic intensity is tailored according to global bleeding and coronary ischemic risk, with longitudinal monitoring allowing bleeding-driven de-escalation or DRT-driven escalation.

The proposed algorithm is built on three progressive strata as seen in Figure 2.

What defines this dynamic approach is the integration of predefined triggers for therapy modification.

In this framework, therapeutic decisions are not fixed but are continuously guided by the patient’s clinical evolution, with particular attention to early signals of instability.

A reduction in hemoglobin represents a key trigger for treatment modification. Specifically, if hemoglobin decreases by more than 2 g/dL between two consecutive measurements, or falls below 9 g/dL, this should be interpreted as a clinically relevant warning sign of ongoing or impending bleeding. In such cases, an immediate de-escalation of antithrombotic therapy is warranted: dual antiplatelet therapy should be reduced to single antiplatelet therapy, and, if the patient is already on single therapy, treatment should be discontinued altogether. In this context, bleeding risk must take absolute priority, and early intervention is essential to prevent progression toward major hemorrhagic events.

Conversely, the detection of device-related thrombosis requires an opposite approach. If imaging follow-up identifies DRT, antithrombotic therapy should be promptly intensified, according to current clinical practice, typically with the introduction of a dedicated therapeutic regimen aimed at thrombus resolution. This bidirectional strategy—rapid de-escalation in response to bleeding signals and timely escalation in the presence of thrombosis—represents the core of a dynamic and patient-centered management model after LAAO.

It is important to underline that the proposed algorithm applies primarily to patients in whom PCI has not been performed or was performed more than 6 months before LAAO. In this setting, the thrombotic risk related to coronary stents is no longer the dominant driver of therapy, allowing a bleeding-oriented strategy to prevail.

Conversely, in patients with recent PCI, antithrombotic management should primarily follow established PCI pathways. In particular, in HBR patients:

- In ACS, a minimum of 6 months [Table S1] of DAPT remains the reference standard.
- In CCS, short DAPT strategies (as short as 1 month) may be considered, especially in high-bleeding-risk patients.

In these cases, the LAAO-related strategy must be integrated within the broader PCI-driven framework, rather than replacing it.

## 7. Conclusions and Future Perspective

In patients with atrial fibrillation and concomitant coronary artery disease undergoing LAAO, post-procedural antithrombotic management remains challenging because thrombotic and bleeding risks coexist and evolve over time. Current evidence supports an individualized approach that considers bleeding risk [35,36], coronary presentation and timing of PCI, together with the transient risk of device-related thrombosis after LAAO. In patients with recent PCI or ACS, established coronary antiplatelet strategies should remain the primary therapeutic framework, whereas simplified antithrombotic regimens may be considered in patients without recent coronary intervention and at high bleeding risk. However, evidence supporting specific regimens in this population remains limited and largely observational. Therefore, the proposed risk-stratified, time-dependent approach should be regarded as a pragmatic decision-support framework rather than a prescriptive treatment strategy. Further prospective studies are needed to define the optimal antithrombotic management in this complex population.

## Data Availability

No new data were created or analyzed in this study. Data sharing is not applicable to this article.

## Supplementary Materials

The following supporting information can be downloaded at: https://www.mdpi.com/article/10.3390/jcm15187181/s1, Table S1: Proposed antithrombotic strategies after left atrial appendage occlusion according to concomitant coronary artery disease clinical scenario and time from the procedure. Treatment intensity and duration are tailored according to the presence and timing of PCI or ACS, balancing ischemic protection with bleeding risk across the early, intermediate, and long-term phases after LAAO.
