# Supplementary material for: Coronary Artery Disease and Atrial Fibrillation in HighBleeding Risk: Indications and Optimal Antithrombotic Therapy After Percutaneous Left Atrial Appendage Occlusion
Source: J Clin Med. 2026 Sep 16;15(18):7181. doi: 10.3390/jcm15187181 (PMC13607074; doi:10.3390/jcm15187181)
Supplement: Supplementary file 1 [file jcm-15-07181-s001.zip › jcm-4491919-supplementary.pdf]

Table S1: Proposed antithrombotic strategies after left atrial appendage occlusion according to concomitant coronary artery disease clinical scenario and time from the procedure. Treatment intensity and duration are tailored according to the presence and timing of PCI or ACS, balancing ischemic protection with bleeding risk across the early, intermediate, and long-term phases after LAAO.

Clinical Scenario	Post-LAAO (0–45/90 days)	Intermediate (45/90 days–6 months)	Long-term (>6 months)
**CCS, no recent PCI** ***(Regimen: LAAO first)***	Ld-DOAc + ASA or DAPT (A+C)	DAPT (aspirin + clopidogrel) or Ld-DOAc monotherapy	ASA monotherapy
**CCS, recent PCI (≤6 mo)** ***(Regimen: balanced)***	DAPT (A+C) consider C discontinuation after 1 mo	ASA monotherapy	ASA monotherapy
**CCS, recent PCI (6-12 mo)** ***(Regimen: LAAO first)***	Ld-DOAc w/o ASA or DAPT	ASA monotherapy	ASA monotherapy
**Recent ACS (w/o PCI) ≤3 mo** ***(Regimen: CAD first)***	DAPT (A+T) or consider de-escalation to A+C after 1 mo	ASA monotherapy after 3 mo or Ticagrelor monotherapy may be considered	ASA monotherapy
**Recent ACS (w/o PCI) 4-12 mo** ***(Regimen: balanced)***	Ld-DOAc + ASA or shortened DAPT (A+C for 6 weeks)	ASA monotherapy	ASA monotherapy

Abbreviations: A= aspirin; ACS= acute coronary syndrome; C= clopidogrel; CAD= coronary artery disease; CCS= chronic coronary syndrome; DOAc= direct oral anticoagulant; LAAO= left atrial appendage occlusion; Ld= low dose; PCI= percutaneous coronary intervention; T= ticagrelor

References

1. Antithrombotic Therapy for Patients Undergoing Cardiac Electrophysiological and Interventional Procedures: JACC State-of-the-Art Review. Journal of the American College of Cardiology. 2024. Di Biase L, Lakkireddy DJ, Marazzato J, et al.Review
2. Antithrombotic Therapy After Left Atrial Appendage Occlusion in Patients With Atrial Fibrillation. Journal of the American College of Cardiology. 2022. Freeman JV, Higgins AY, Wang Y, et al.
3. Network Meta-Analysis of Initial Antithrombotic Regimens After Left Atrial Appendage Occlusion. Journal of the American College of Cardiology. 2023. Carvalho PEP, Gewehr DM, Miyawaki IA, et al.

4. Antithrombotic Therapy in High Bleeding Risk, Part I: Percutaneous Cardiac Interventions. JACC. Cardiovascular Interventions. 2024. Galli M, Gragnano F, Berteotti M, et al.
5. Short-Term Anticoagulation Versus Dual Antiplatelet Therapy for Preventing Device Thrombosis Following Left Atrial Appendage Closure: The ANDES Randomized Clinical Trial. Circulation. 2025. Rodés-Cabau J, Nombela-Franco L, Cruz-Gonzalez I, et al.
6. Anticoagulation Alone vs Anticoagulation Plus Aspirin or DAPT Following Left Atrial Appendage Occlusion. Journal of the American College of Cardiology. 2024. Reinhardt SW, Gibson DN, Hsu JC, et al.
